# The association between preoperative modified frailty index and postoperative complications in Chinese elderly patients with hip fractures

**DOI:** 10.1186/s12877-021-02330-7

**Published:** 2021-06-16

**Authors:** Yanjiao Shen, Qiukui Hao, Yuting Wang, Xiaoyan Chen, Jiaojiao Jiang, Birong Dong, Gordon Guyatt

**Affiliations:** 1 The Center of Gerontology and Geriatrics/National Clinical Research Center of Geriatrics, West China Hospital, Sichuan University, #37 Guoxuexiang, Chengdu, 610041 Sichuan China; 2 Department of Health Research Methods, Evidence and Impact, McMaster University, Hamilton, Canada; 3 The Center of Rehabilitation, West China Hospital, Sichuan University, Chengdu, Sichuan China

**Keywords:** Frailty, Medical records, Postoperative complications, Hip fracture

## Abstract

**Objectives:**

To investigate the role of a preoperative modified frailty index (mFI) based on data from medical records in predicting postoperative complications among older Chinese patients with hip fractures.

**Methods:**

This retrospective cohort study included consecutive older patients with hip fracture admitted to the Department of Orthopaedics, West China Hospital, Sichuan University, from December 2010 to June 2017 who underwent surgical repair. We selected 33 variables, including characteristics of hip fracture, to construct a mFI. Each variable was coded with a value of 0 when a deficit was absent or 1 when it was present. We calculated the mFI as the proportion of positive items and defined frailty as mFI value greater than or equal to 0.21 according to threshold proposed by Hoover et al. We examined the relationship between mFI and severity of postoperative complications and the occurrence of in-hospital pneumonia including statistical adjustment for several demographics (e.g. age, gender, and marital status) and habits (smoking and alcohol intake), time from fracture to surgery in the multivariable model.

**Results:**

We included 965 patients (34% male; mean age: 76.77 years; range: 60 to 100 years) with a prevalence of frailty of 13.06%. The presence of frailty was associated with a higher severity of complications (OR: 2.07; 95% CI: 1.40 to 3.05). Frail patients were more likely to develop in-hospital pneumonia than non-frail patients (OR: 2.08; 95% CI: 1.28 to 3.39).

**Conclusion:**

The preoperative modified frailty index based on data from medical records proved significantly associated with postoperative complications among older patients with hip fractures undergoing hip surgery.

**Supplementary Information:**

The online version contains supplementary material available at 10.1186/s12877-021-02330-7.

## Background

Hip fracture is a common type of fracture in the older people. According to population dynamics forecast, the incidence of hip fracture will triple by 2050 worldwide (reaching 6.3 million cases) [1]. Hip fractures not only reduce the quality of life for the older people but also incur a heavy financial burden on society [2].

For most hip fractures, surgery is the best treatment [3]. However, older patients with hip fracture often suffer from chronic diseases and reduced ability to cope with the physiologic stress associated with the fracture. Frailty refers to a geriatric state or syndrome that reflects the body’s decline in physiological reserve and ability to deal with stress and a consequent increase in vulnerability [4]. Previous studies found that frailty is a good predictor of postoperative outcomes in older patients undergoing hip surgery [5, 6]. However, the extent of the variety of the frailty measures that could accurately predict postoperative complications in older hip fracture patients remains uncertain [7, 8].

The Frailty index (FI) is the ratio of health defects present in the older patients to all measured variables based on the deficits accumulation theory [4]. The FI does not rely on specific variables included and integrates multiple complex health information into a single index with a simple mathematical model, which can better reflect the overall health. Previous studies found that FIs or modified FIs (using 5 to 51 variables) were able to predict adverse outcomes in hip fracture patients [8–11]. However, all of these studies were conducted in western countries.

Both the medical care model and patients’ characteristics (race, socioeconomic factor et al) are substantially different between China and Western countries. The role of FI in predicting postoperative outcomes in Chinese older patients with hip fracture is not well understood. Furthermore, previous studies found that characteristics of presentation of hip fracture were related to postoperative outcomes [12]. Therefore, we used data from medical records to construct a 33-item mFI including variables describing presentation characteristics of hip fracture to determine its potential role in predicting postoperative complications among Chinese older patients with hip fracture.

## Methods

### Study design and participants

This study was a retrospective cohort study, including all consecutive older patients admitted to the Department of Orthopedics, West China Hospital of Sichuan University from December 2010, up to June 2017. All patients were diagnosed as having a hip fracture and treated with a surgical operation. The medical research ethics committee of West China Hospital, Sichuan university approved this study (No. 2018-95). All methods were in accordance with relevant guideline and regulations in this present study.

Patients who met the following criteria were eligible: age of 60 years or older; admitted to West China Hospital of Sichuan University due to hip fracture (physician or orthopedist made the diagnosis) from December 2010 to June 2017, received a surgical operation in the hospital. We excluded the following patients: those with any bone tumours, medical record or surgical records were not accessible (e.g., incomplete or confidential records). For a given patient who had been hospitalized or received surgical treatment more than one time for hip fracture, we included the latest hospital admission. We collected these data from the inpatient Hospital Information System of West China Hospital.

### Construction of modified frailty index

Following previously articulated principles [13], we selected health-related variables available in the medical record to construct the mFI. The variables we chose generally increase in frequency with age (except the hip fracture characteristic variables), do not saturate too early, biologically sensible and missing data must be less than 5% of the study population. The chosen variables include diseases, symptoms, and physical examination findings cover a range of systems and are all associated with health status. We also selected the variables describing presentation characteristics of the hip fractures including hip deformity, local swelling type of wound and incision, shortening, angular, separation or rotation displacement to include in the mFI (Additional file 1).

Each variable contained in the mFI was coded with a value of 0 when a deficit was absent and 1 when it was present. If there were missing data, we removed the variable from both the denominator and numerator for that patient. We calculated the mFI according to the following formula mFI = the total number of deficits / the total number of variables considered (here, 33 when all variables were available). For example, if we considered 33 potential deficits, and 15 were present on a given person, their mFI would be 15/33 = 0.45. The higher mFI indicates the patient has had more health defects and is frailer.

### Outcomes

Our outcomes were all postoperative complications of surgery for hip fracture and in-hospital pneumonia. Orthopedic surgeons or physicians made the diagnosis of these postoperative complications. We used a well-described classification system (Clavien–Dindo 2004) to classify postoperative complications into severity grades I, II, III and above [14]. The timeframe of the outcome is any time during the hospital stay after surgery.

### Quality control

We collected all these variables and outcomes from the electronic medical record system of the hospital through the following two steps: first, the staff in the health information department downloaded the medical records of all eligible patients without patients’ identity. Second, two research physicians extracted data and, where necessary, made a judgment for every variable. For example, if the patient had diabetes, the diabetes variable would be code 1, if the patient without diabetes, the diabetes variable would be code 0. If any of the research physicians found the data that were not clear, they checked the data in the paper-version medical record and if necessary, discussed their uncertainties with the whole team. This occurred most often with the following variables: “appetite”, “sleep quality”, “weight change (loss/increase)”, “abnormality of stool” or “abnormality of urine” for few digital medical records was not complete downloaded. We collected the variable to construct the mFI according to the assessment within 24 h of hospital admission.

### Statistical analysis

We performed statistical analysis using the SPSS 26.0 statistical package (IBM Corp., Somers, NY). We used quantiles, percentages, means, and standard deviations (SDs) according to the types of variables. As appropriate, we used chi-square tests, t-test, and Wilcoxon rank-sum test to test the statistical difference between frailty and non-frailty groups. We used the Spearman rank correlation to test the relationship between FI and the severity grades of all postoperative complications. We used multivariable analysis (ordinal logistic regression for the severity grades of all postoperative complications and binary logistic regression for the in-hospital pneumonia outcome) to adjust the associated confounding factors and calculated the OR value of each factor and the corresponding 95% confidence interval (95% CI). We adjusted the following confounding variables, age, gender, marital status and lifestyle variables (smoking, alcohol consumption history), time from fracture to surgery in the multivariable model.

In our study, we treat mFI both as a continuous and binary variable. For the binary context, we defined frailty as mFI value greater than or equal to 0.21, and non-frailty as less than 0.21 according to threshold point proposed by Hoover et al. among community-dwelling older people [15]. The cut-off value also are applied to patients with fibrotic interstitial lung disease [16], acute respiratory illness [17] and patients after primary and revision total hip arthroplasty [11]. For continuous context, we explore the role of mFI increased by 0.01 or by per standard deviation in predicting the interested outcomes using the Logistic regression model. We used a threshold two-sided *P* value of 0.05 to define statistical significance.

## Results

### Baseline characteristics

From December 2010 to June 2017, 968 patients over 60 years old admitted to West China Hospital of Sichuan University underwent hip fracture surgery, 3 cases with hip fracture and malignant tumor were excluded, finally, 965 patients were included. In this cohort, the mean age of the patients (range) was 76.77 years (60 - 100 years), of which the majority were females (66%) and had a duration of time between facture and surgery of more than 48 h. See the details of included patients’ characteristic in Table 1.

**Table 1:** Demographic and clinical characteristics of the cohort

Characteristics	Total (***n*** = 965)	Non-frailty (***n*** = 839)	Frailty (***n*** = 126)	χ2/T	P
**Age (mean ± SD)years**	76.77 ± 8.76	76.75 ± 8.70	76.93 ± 9.19	−0.21	0.83
**Age**				0.01	0.91
60-70 years	241 (25.00%)	209 (86.72%)	32 (13.28%)		
> 70 years	724 (75.00%)	630 (87.02%)	94 (12.98%)		
**Gender**				2.49	0.11
Male	328 (34.00%)	293 (89.33%)	35 (10.67%)		
Female	637 (66.00%)	546 (85.71%)	91 (14.29%)		
**Smoking**				0.09	0.76
No	813 (84.20%)	708 (87.08%)	105 (12.92%)		
Yes	152 (15.80%)	131 (86.18%)	21 (13.82%)		
**Alcohol intake**				0.70	0.40
No	849 (88.00%)	741 (87.28%)	108 (12.72%)		
Yes	116 (12.00%)	98 (84.48%)	18 (15.52%)		
**Marital status**				0.29	0.59
Married	753 (78.00%)	657 (87.25%)	96 (12.75%)		
Divorced / widowed	212 (22.00%)	182 (85.85%)	30 (14.15%)		
**Time from fracture to admission**				1.28	0.26
≤ 48 h	618 (64.00%)	543 (87.86%)	75 (12.14%)		
> 48 h	347 (36.00%)	296 (85.30%)	51 (14.70%)		
**Time from fracture to surgery**				1.42	0.23
≤ 48 h	61 (6.30%)	50 (81.97%)	11 (18.03%)		
> 48 h	904 (93.70%)	789 (87.28%)	115 (12.72%)		

Of the 965 hospitalized older patients with hip fracture, 126 (13.06%) were in the frailty group. Female and male had a similar frailty prevalence rate (14.29% vs. 10.67%, respectively, χ2 = 2.49, *P* = 0.11). The mFI score median (range) was 0.12 (0-0.39). The distribution of mFI scores is shown in the figure s1. We did not find statistically significant differences in age, smoking, alcohol intake, marital status, and time from fracture to surgery between the frailty group and the non-frailty group (Table 2**)**.

**Table 2:** Frailty status and postoperative complications in hospitalized elderly patients with hip fracture

	Non-frailty (***n*** = 839)	Frailty (***n*** = 126)		
**Univariate analysis**			**χ2/Z**	P
**Clavien-Dindo classification (2004)**			16.86	0.001
No complications	652 (77.7%)	78 (61.9%)		
Grade I	63 (7.5%)	14 (11.1%)		
Grade II	96 (11.4%)	29 (23.0%)		
Grade III and above	28 (3.3%)	5 (4.0%)		
**In-hospital pneumonia**			9.24	0.002
No	746 (88.9%)	100 (79.4%)		
Yes	93 (11.1%)	26 (20.6%)		
**Adjusted Multivariate analysis**^**a**^				
	**B**	**S.E.**	**Wald**	**OR(95%CI)**
**Clavien-Dindo classification (2004)**	0.73	0.20	13.48	2.07 (1.40-3.05)
**In-hospital pneumonia**	0.73	0.25	8.60	2.08 (1.28- 3.39)

^a^ adjusted the age, gender, marital status, smoking, alcohol intake, time from fracture to surgery

### The severity grades of all postoperative complications

A greater proportion of patients without frailty than those with frailty remained free of complications. In contrast, for each group with complications, the proportions with frailty was larger than the proportions of those without frailty (Table 2**)**. The mFI was thus positively associated with the severity grades of all postoperative complications (Spearman’s r = 0.19, *P* < 0.001). In the univariable analysis, there was a statistically significant difference in the Clavien-Dindo classification between frailty and non-frailty patients (χ2 = 16.86, *P* = 0.001) (Table 2). In the multivariable analysis, we found that the odds of being higher level Clavien-Dindo grade was higher in those with frailty than those without frailty (adjusted OR: 2.07; 95% CI: 1.40 to 3.05, *P* < 0.001) (Table 2). We also found that each increase of a standard deviation of mFI, the risk of severity grades of all postoperative complications increased by 1.57 times, 95% CI (1.35 to 1.82, *P* < 0.001). For an increase in mFI score by 0.01, the risk of severity grades of all postoperative complications increased by 1.07 (95%, 1.04 to 1.09).

### In-hospital pneumonia

Patients with frailty were more likely to develop in-hospital pneumonia than non-frail patients (20.6% vs 11.1%, χ2 = 9.24, *P* = 0.002) (Table 2). The multivariable analysis shows that patients with frailty status statistically increased the risk of in-hospital pneumonia (adjusted OR: 2.08; 95% CI: 1.28 to 3.39, *P* = 0.003) (Table 2**)**. We also found that each increase of a standard deviation of mFI, the risk of in-hospital pneumonia increased by 1.65 times, 95% CI (1.37 to 2.00, *P* < 0.001). For an increase in mFI score by 0.01, the risk of in-hospital pneumonia increased by 1.07 (95%, 1.05 to 1.10).

## Discussion

The main findings were frailty status measured by modified Frailty index considering characteristics of hip fracture significantly associated with postoperative adverse outcomes, including Clavien-Dindo classifications and in-hospital pneumonia among Chinese elderly patients with hip fractures.

This study is the first study to apply a mFI with characteristics of hip fracture to the perioperative risk assessment of Chinese elderly patients with hip fracture. As in other populations, hip fracture patients were more often women than men (66% vs. 34%) [8–10]. Our study had a representative population in terms of distribution of sex. Construction of the mFI did not include the same items as its original validated version due to lack of comprehensive geriatric assessment data [13, 18–21], we selected the most objective variables (chronic diseases and variables describing characteristics presentation of hip fracture) to construct the mFI in the present study. To include the hip fracture characteristic variables may be one of the limitations, but it can also be one of our study’s strengths. Here, our data suggested that healthcare providers could be benefited by also taking into account these objective variables in frailty assessment.

The results of our study must be interpreted with caution because of the following limitations. Firstly, we did not have the data on the incidence of delirium, which is a common complication in surgical patients. However, the assessment of delirium requires face-to-face assessment with the patient. This study is a retrospective study, and delirium was not well documented in the medical records. Secondly, over 90% of patients (*n* = 904, 93.7%) in our study missed the best time to surgery window recommended by current guidelines (48 h) [22], which may not represent patients who had hip fracture surgery within the appropriate surgery time window. Thirdly, we did not have a mortality data and did not conduct a prospective follow-up study to validate further and modify the role of mFI in predicting mortality and long-term postoperative adverse outcomes in older patients undergoing hip surgery. Fourthly, we have small number of patients (less than 5% of participants) with missing data in some variables in frailty index. This small number of missing data is likely to influence the number of our results but will not change our conclusions. Furthermore, to impute missing data in the mFI construction may introduce measurement error and bias [23]. Therefore, we did not impute the missing data and perform further sensitivity analysis.

The results of our study are consistent with those of previous studies in terms of mFI in predicting postoperative adverse outcomes [6, 9–11] . In a recent study conducted by Johnson et al., the researcher also used data from the electronic medical record to construct 32-FI and found that the preoperative mFI associated with perioperative complications and 1-year mortality following primary and revision total hip arthroplasty surgery [11]. The study conducted by Dayama et al. found that 11-item FI was associated with complications and mortality among patients undergoing hip arthroplasty surgery [9]. Traven et al. also found that even a 5-item modified FI also could be an independent predictor of postoperative mortality and morbidity in patients with hip fracture undergoing surgery [10]. All these studies were conducted in the United States. Our study extends the findings to Chinese older patients undergoing hip surgery. One study conducted in Taiwan used clinical frailty scale to measure frailty also yielded similar results with the present study [24]. Therefore, we should pay attention to the preoperative assessment of frail elderly patients with hip fracture.

## Conclusion

The modified Frailty index based on data from medical records, including variables describing characteristics presentation of hip fracture, proved significantly associated with the postoperative complications among Chinese older patients with hip fracture undergoing hip surgery. Preoperative assessment of frailty may provide useful prognostic information for older patients with hip fracture undergoing hip surgery.

## Supplementary Information

**Additional file 1.**

## Data Availability

All data generated or analysed during this study are included in this published article and its supplementary information files.

## Abbreviations

FI: Frailty index
OR: Odds ratio
SD: Standard deviation
CI: Confidence interval
