# Supplementary material for: The association between preoperative modified frailty index and postoperative complications in Chinese elderly patients with hip fractures
Source: BMC Geriatr. 2021 Jun 16;21:370. doi: 10.1186/s12877-021-02330-7 (PMC8207648; doi:10.1186/s12877-021-02330-7)
Supplement: Supplementary file 1 — Additional file 1. [file 12877_2021_2330_MOESM1_ESM.docx]

**Appendix 1: Overview of the variables included in the modified frailty index**

| **Number** | **Deficits** | **Values** |
| --- | --- | --- |
| **1** | Diabetes | No = 0, yes = 1 |
| **2** | Endocrine system diseases other than diabetes | No = 0, yes = 1 |
| **3** | Hypertension | No = 0, yes = 1 |
| **4** | Coronary atherosclerotic heart disease | No = 0, yes = 1 |
| **5** | Cardiovascular diseases other than coronary atherosclerotic heart disease | No = 0, yes = 1 |
| **6** | Chronic obstructive pulmonary disease | No = 0, yes = 1 |
| **7** | Respiratory diseases other than chronic obstructive pulmonary disease | No = 0, yes = 1 |
| **8** | Osteoporosis | No = 0, yes = 1 |
| **9** | Cerebral infarction / stroke | No = 0, yes = 1 |
| **10** | Nervous system diseases other than cerebral infarction / stroke | No = 0, yes = 1 |
| **11** | Hepatobiliary disease | No = 0, yes = 1 |
| **12** | Digestive system diseases other than hepatobiliary disease | No = 0, yes = 1 |
| **13** | Kidney disease | No = 0, yes = 1 |
| **14** | Urinary system diseases other than kidney disease | No = 0, yes = 1 |
| **15** | Osteoarthrosis | No = 0, yes = 1 |
| **16** | Tumor | No = 0, yes = 1 |
| **17** | Venous thrombosis | No = 0, yes = 1 |
| **18** | Mental system disease | No = 0, yes = 1 |
| **19** | Current mental state | No = 0, yes = 1 |
| **27** | Dizzy | No = 0, yes = 1 |
| **20** | Appetite | No = 0, yes = 1 |
| **21** | Sleep quality | No = 0, yes = 1 |
| **22** | Weight loss / increase | No = 0, yes = 1 |
| **23** | Abnormality of stool | No = 0, yes = 1 |
| **24** | Abnormality of urine | No = 0, yes = 1 |
| **25** | Hip deformity | No = 0, yes = 1 |
| **26** | Local swelling | No = 0, yes = 1 |
| **28** | Open wound | No = 0, yes = 1 |
| **29** | Type of incision | No = 0, yes = 1 |
| **30** | Shortening displacement | No = 0, yes = 1 |
| **31** | Angular displacement | No = 0, yes = 1 |
| **32** | Separation displacement | No = 0, yes = 1 |
| **33** | Rotation displacement | No = 0, yes = 1 |


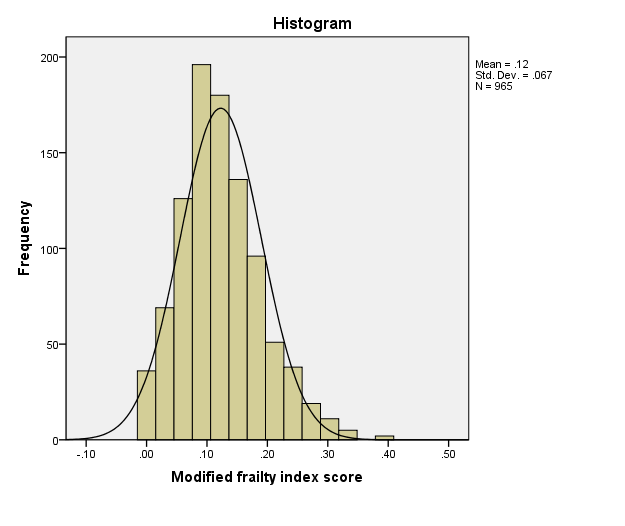


**figure s1** Distribution of the frailty index score
